# THE ASSOCIATION OF DAILY URINARY AND BOWEL INCONTINENCE WITH EMOTIONAL FUNCTIONING IN YOUNG ADULTS WITH SPINA BIFIDA: AN ECOLOGICAL MOMENTARY ASSESSMENT STUDY

**DOI:** 10.2340/jrm.v58.45594

**Published:** 2026-06-17

**Authors:** Devon J. HENSEL, Audrey I. YOUNG, Konrad M. SZYMANSKI

**Affiliations:** 1 Division of Adolescent Medicine, Department of Pediatrics, Indiana University School of Medicine, Indianapolis; 2 Department of Biology, DePauw University, Greencastle, Indiana; 3 Division of Pediatric Urology, Riley Hospital for Children at Indiana University Health, Indianapolis, IN, USA

**Keywords:** spina bifida, health diary, urinary incontinence, bowel incontinence

## Abstract

**Objective:**

Urinary and bowel incontinence are common in young adults with spina bifida and may affect emotional functioning and health-related quality of life. Prospective data describing day-level incontinence experiences in this population are limited. This study used ecological momentary assessment to characterize daily variability in urinary and bowel incontinence and to examine associations with mood, incontinence-related anxiety, and health-related quality of life.

**Methods:**

In a 30-day ecological momentary assessment study of adults with spina bifida and recent incontinence, young adults with spina bifida (*n* = 23; ages 18–26) completed 643 end-of-day assessments. Daily measures included urinary and bowel incontinence frequency, amount, dry intervals, management independence, activity avoidance, positive and negative mood, and incontinence-related anxiety. Health-related quality of life was assessed at study completion. Analyses were exploratory and used nonparametric methods appropriate for ordinal and intensive repeated measures data.

**Results:**

Urinary incontinence was reported on 54.1% of study days and bowel incontinence on 20.8%. For urinary incontinence, greater frequency, shorter dry intervals, larger amounts, and activity avoidance were associated with lower positive mood, higher negative mood, and greater urinary incontinence-related anxiety. For bowel incontinence, greater symptom severity, management dependence, and activity avoidance were associated with poorer mood and higher bowel incontinence-related anxiety. Multiple day-level incontinence characteristics and daily emotional responses were associated with lower end-of-month health-related quality of life.

**Conclusion:**

In young adults with spina bifida, day-level incontinence burden is associated with same-day emotional functioning and longer-term health-related quality of life. These findings highlight the relevance of day-level symptom variability for rehabilitation assessment and individualized continence management.

## LAY ABSTRACT

Many young adults with spina bifida experience bladder and bowel leakage, but clinic visits often rely on people remembering and summarizing their symptoms over long periods. In this study, we asked participants to report their experiences each day for one month using simple phone-based check-ins. We found that leakage does not affect every day in the same way. On days when symptoms were more frequent or harder to manage, participants reported worse mood, more worry, and were more likely to avoid activities. These day-to-day experiences also related to overall quality of life at the end of the month. For clinical practice, this means that “average” symptom reports may miss important patterns. Tracking symptoms in real time can help clinicians and patients better understand what is happening in daily life and tailor treatment plans to support independence, participation, and well-being.

Spina bifida (SB) is a non-progressive congenital anomaly caused by spinal cord and vertebral malformation during embryonic development (1). Individuals with SB frequently experience lifelong secondary impairments, including neurogenic bladder and bowel dysfunction (2). Among young adults with SB (YASB), past-month urinary incontinence (UI) prevalence is estimated at between 50% and 75%, and bowel incontinence (BI) between 41% and 55%, with many reporting co-occurring symptoms (3, 4). UI and BI restrict participation in everyday activities (5), reduce functional independence (6), and contribute to clinically meaningful declines in health-related quality of life (HRQoL) (7, 8).

Advances in pediatric and rehabilitative care have enabled most individuals born with SB to reach adulthood (9), reframing continence management as a long-term rehabilitation priority rather than solely as a pediatric concern. Emerging adulthood introduces increasing expectations for independent health self-management and sustained participation in educational, vocational, and social roles (10, 11). For young adults with SB (YASB), urinary and bowel incontinence disrupt daily routines and activities, creating barriers to long-term autonomy at a stage when independence and participation are actively being established (6,12). How continence is managed is therefore closely tied to independence and HRQoL outcomes in this population (13,14). Yet the “in the moment” data needed to understand how incontinence unfolds in daily life remain largely absent from current clinical assessment.

Current clinical practice relies heavily on retrospective symptom summaries to guide continence care (12). YASB are typically asked to compress complex incontinence experiences into a small number of indicators (e.g., leakage severity, number of dry intervals) recalled across extended time frames such as “in the past month” or “since the last clinic visit” (4). Although pragmatic given the difficulty of directly observing incontinence, these summaries are vulnerable to recall bias and obscure meaningful between-person differences in how UI and BI impact everyday functioning (13–16). Two individuals reporting identical incontinence symptom frequency may experience markedly different real-life functional consequences, yet standard assessments often treat these reports as equivalent, encouraging uniform “one size fits all” treatment strategies that may not address individual needs. Both clinical experience and research data suggest that incontinence varies not only from person to person but also within the same person over time (4, 17–21). Despite this variability, fundamental questions relevant to clinical decision-making remain unanswered, including: *how often* do UI and BI occur for YASB in day-to-day life? How does the *context* in which they occur shape their *functional impact*? Do these relationships *differ* between UI and BI? Addressing these questions requires temporally proximal data capable of capturing incontinence as it occurs in everyday life (22).

In the present study, we used ecological momentary assessment (EMA) to generate these data. EMA is a prospective, person-centred measurement approach that improves accuracy by capturing repeated, “in-the-moment” reports of symptoms (e.g., frequency, amount, dry intervals) and the context in which they occurred (e.g., feeling negatively about incontinence, any activity avoidance, self-management) (23, 24). Data are collected on a pre-specified schedule (e.g., once daily) (25) over extended periods (e.g., weeks or months) (26, 27), allowing typical incontinence patterns to be observed in everyday life. Over time, these repeated observations describe how incontinence presents in daily functioning for YASB (28). EMA methods are well established in health behaviour research (for excellent reviews, see Perski et al., 2022 [29] and Morris et al., 2020 [30]) and we have begun to use this method with individuals in the SB population (4, 17, 21, 31).

Accordingly, the primary objectives of our paper were to examine:

1. the daily frequency (e.g., how often) and characteristics (e.g., amount, dry intervals, affect or activity avoidance) of daily UI and BI among YASB; and
2. the associations between daily UI and BI frequency and characteristics with daily mood and daily incontinence anxiety.

As an exploratory aim, we examined whether daily incontinence experiences, mood, and anxiety were associated with end-of-study HRQoL.

## METHODS

### Study design and participants

Data were drawn from a larger 30-day study (R21DK121355) (4, 17) that examined the feasibility of using ecological momentary assessment (EMA) to describe daily incontinence experiences among adults with SB. Participants were recruited through social media (e.g., researcher or SB association posts), clinical referral, or through completed participant referral. Study eligibility requirements included being 18 years or older, having SB, having any UI and/or BI in the past 4 weeks, having English language literacy, and having normal to mildly impaired cognitive development. As part of this larger study, participants completed 30 end-of-day EMAs that assessed UI and/or BI prevalence and episode-specific characteristics, any UI or BI anxiety, and daily mood. All participants in the larger study provided written informed consent to participate in this study. This project was approved by the Institutional Review Board of Indiana University (#1907916729) and achieved excellent retention (97.5%), EMA completion (95.8%), and data accuracy (98.6%) and acceptability (> 90% enjoyed the study, found the protocol easy, felt supported, and would participate again). Raw data and codebooks are available within the Open Science Framework (32).

For the current paper, we drew an analytic sample of YASB participants aged 18–27 years (*n* = 23 [26.1% of all study participants [*n* = 88]). They were mostly female (78.3%), White (91.3%), and heterosexual (82.6%) and had a median educational completion of some college. Half reported their relationship status to be single and about a third were working part- or full-time. More than half of YASB reported having a ventriculoperitoneal shunt (VPS), and about 40% were community ambulators. A third (39.1%) lived independently while most (60.1%) lived with 1 or more parents. All reported some kind of incontinence in the 4 weeks prior to the study (17.3% UI only, 13.0% reported BI only; 70% both UI and BI).

### Measures

*Daily incontinence variables*. For both UI and BI, we assessed *frequency* (original item: once, twice, 3–5 times, 6 or more times; item trichotomized for this work based on low frequencies at the higher end: once, twice, vs 3 or more times), *volume* (small [a little bit], medium, large [a lot]), *length of dry intervals* (more than 4 h, less than 4 h, never dry/always soiled), *any activity avoidance* (no/yes), and *incontinence management* (needed some help vs fully on own).

*Outcome variables*. We used 3 variables, including daily *positive mood* (“happy” and “friendly”), *negative mood* (“angry” and “irritable”) (PANAS; both additive indices of the 2 x 5-point Likert items [not at all to extremely]) (33), and *incontinence anxiety* (single Likert- type item: “To what extent did you feel anxious about [incontinence type] today?” (not at all or very slightly, a little, moderately, extremely; range: 1–5, 5 representing highest anxiety).

### Statistical analysis

Our primary goal was descriptive and exploratory, focusing on identifying clinically meaningful patterns rather than estimating causal effects. We used descriptive statistics to characterize the daily frequency and characteristics of daily UI and BI. Owing to the ordinality of mood and anxiety variables, and to maintain interpretability in this preliminary study, we used nonparametric Wilcoxon rank-sum tests to examine median differences in positive mood, negative mood, and incontinence anxiety across UI and BI characteristics (34). Finally, we used Spearman’s rank coefficient to assess the correlation between daily incontinence characteristics, daily mood and incontinence anxiety, and HRQoL at the end of the study (Objective 3) (35). All analyses were performed in Stata (v. 19.0; StataCorp LLC, College Station, TX, USA) (36).

## RESULTS

### Overall daily UI and BI patterns

As indicated in Table I, about 6 out of every 10 days were associated with a report of any type of incontinence (53.6%; 370/690). Considering only days with incontinence, 63.7% (236/370) were UI only. About 1 in 10 (9.3%: 22/370) were BI only, and a third (30.2%: 112/370) had UI and BI. UI was reported on 50.4% (348/690) of all study days, while BI was noted on 19.4% (134/690) of all study days (*p* < 0.001). Sensitivity analyses suggested that neither the general occurrence of incontinence (*p* = 0.826) nor the type of incontinence was more likely to occur on any given day of the week as compared with others (UI: *p* = 0.272; BI: *p* = 0.055). Prior analysis also affirm that UI or BI reporting did not change as a function of study participation (4).

**Table I:** Background characteristics and daily incontinence frequency of young adults with spina bifida (YASB) (*n* = 23)

Background characteristic	
Age, mean (SD); median	22.9 (2.31); 23.0
Birth-assigned sex (female), %	78.3
Gender identity (female), %	78.3
Sexual identity orientation, %	
Heterosexual	82.6
Gay or lesbian	0
Bisexual	13
Asexual	4.3
Something else	0
Race/ethnicity, %	
White	91.3
Black	8.7
American Indian or Alaska Native	0.0
Asian	0.0
Other	0.0
Multiple races	0.0
Hispanic or Latino (yes), %	4.4
Annual household income, %	
< $20,000	20.0
$20,000 to $39,000	15.0
$40,000 to $59,000	10.0
$60,000 to $79,000	5.0
$80,000 to $100,000	5.0
$100,000 or more	15.0
Unsure	30.0
Highest education level completed, %	
Less than high school	0.0
High school degree or equivalent	39.3
Some college but no degree	17.3
degree	34.7
Graduate degree	8.0
Current work status, %	
Working full time	17.3
Working part time	26.1
Unemployed and looking for work	17.3
Unemployed and not looking for work	13.0
On disability	26.1
Currently attending school (full or part time), %	17.3
Current relationship status, %	
Single and not dating	56.5
Single and dating but not in a relationship	8.7
In a relationship, but not living together	13.0
In a relationship, living together (not married)	8.7
Married	13.0
Divorced	0.0
Widowed	0.0
Currently living independently (yes), %	39.3
Ambulation, %	
I move only in my wheelchair	26.1
I walk only during therapy. I use a wheelchair the rest of the time	4.3
I walk inside and outside (with or without a walker, braces or crutches). I only use a wheelchair for long trips.	17.4
I walk inside and outside without any help	26.1
Have a ventriculoperitoneal shunt (VPS) (yes), %	60.8
Any incontinence at enrolment (past 4 weeks), %	100.0
Urinary incontinence only	17.4
Bowel incontinence only	13.0
Both urinary incontinence and bowel incontinence	69.6
Catheterize bladder to drain urine, %	87.1
Malone antegrade colonic enema (MACE) to empty bowels, %	18.8
Daily incontinence (*n* = 643 EMA entries)	
Any incontinence (*n* = 370), %	57.5
Urinary incontinence only (*n* = 236)	63.7
Bowel incontinence only (*n* = 22)	9.3
Both urinary incontinence and bowel incontinence (*n* = 112)	30.2
Any urinary incontinence, %	54.1
Any bowel incontinence, %	20.8
Urinary incontinence anxiety, %	
Not at all	56.4
A little	20.2
Moderately	10.6
Quite a bit	5.7
Extremely	6.9
Bowel incontinence anxiety, %	
Not at all	46.2
A little	14.9
Moderately	2.9
Quite a bit	2.2
Extremely	33.5

### Objective 1: Describe daily UI and BI frequency and characteristics

*Urinary incontinence.* As set out in Table II, the most common UI frequency was once during a day (44.8%). The median dry interval was less than 4 h, although one-third of UI events were dry for more than 4 h. The median (and most common) amount of UI reported was a “medium” amount; only 1 in 5 UI days reported a “large” amount of urine. Nearly all (89.8%) UI events required no management help, and very few (7.7%) required activity avoidance. The median daily anxiety when UI occurred was relatively low (1.8; corresponding to between “not at all” and “very slightly”). None of these variables – daily UI frequency (*p* = 0.991), amount (*p* = 0.830), dry intervals (*p* = 0.671), management (*p* = 0.864), avoiding activities (*p* = 0.499), and incontinence negativity (*p* = 0.973) – varied significantly by day of the week.

**Table II:** Daily urinary characteristics, association with daily affect and incontinence anxiety, and association with health-related quality of life among young adults with spina bifida

Item	Overall percentage	Correlation with end-of-study HRQoL	Positive mood		Negative mood		UI anxiety	
			Median (IQR)	Wilcoxon rank sum or Kruskal Wallis test *p*-value	Median (IQR)	Wilcoxon rank sum or Kruskal Wallis test *p*-value	Median (IQR)	Wilcoxon rank sum or Kruskal Wallis test *p*-value
Frequency								
Once	44.83	–0.253***	5.0 (4.0–7.0)	< 0.001	3.0 (2.0–5.0)	.045	1.0 (1.0–2.0)	< 0.001
Twice	16.67		4.0 (4.0–6.0)		4.0 (2.0–6.0)		1.0 (1.0–3.0)	
Three or more times	38.51		6.0 (5.0–8.0)		4.0 (4.0–5.0)		2.0 (1.0–3.0)	
Dry intervals								
More than 4 h	31.32	–0.277***	8.0 (6.0–8.0)	< 0.001	2.0 (2.0–4.0)	< .001	1.0 (1.0–2.0)	.078
Less than 4 h	54.60		6.0 (5.0–8.0)		4.0 (3.0–5.0)		1.0 (1.0–2.0)	
Never dry	14.08		4.0 (4.0–7.0)		4.0 (3.0–5.0)		2.0 (1.0–3.0)	
Amount								
Small	25.00	–0.286***	8.0 (6.0–8.0)	< 0.001	3.0 (2.0–4.0)	.013	1.0 (1.0–2.0)	< 0.001
Medium	55.75		5.0 (4.0–7.0)		4.0 (2.0–6.0)		1.0 (1.0–2.0)	
Large	19.25		4.0 (5.0–7.0)		4.0 (2.0–6.0)		2.0 (1.0–4.0)	
Independent management								
No	16.67	0.066	6.0 (5.0–8.0)	.389	4.0 (3.0–5.0)	.571	2.0 (1.0–3.0)	0.172
Yes	83.33		6.0 (4.0–8.0)		4.0 (2.0–5.0)		1.0 (1.0–2.0)	
Avoided any activities								
No	91.07	–0.168**	6.0 (4.0–8.0)	< 0.001	4.0 (3.0–6.0)	.667	1.0 (1.0–5.0)	0.002
Yes	8.93		4.0 (3.0–6.0)		4.0 (2.0–6.0)		2.0 (2.0–5.0)	

*p < 0.05;

** *p* < 0.01;

*** *p* < 0.001

*Bowel incontinence.* As reported in Table III, BI was most common once per day (82.1%), typically with a small amount of stool leak (52.9%). Half of BI events involved clean intervals of more than 4 h (54.9%). A majority (88.3%) of BI events were managed independently, and 1 in 4 events involved avoiding activities (26.6%). The median daily anxiety when BI occurred was relatively low (2.6; corresponding to “very slightly” to “a little”). None of these variables – daily BI frequency (*p* = 0.297), amount (*p* = 0.485), clean intervals (*p* = 0.670), management (*p* = 0.740), avoiding activities (*p* = 0.703), and FIA (*p* = 0.885) – were different by any specific day of the week.

**Table III:** Daily bowel incontinence characteristics and association with daily affect and incontinence anxiety among young adults with spina bifida

Item	Overall percent	Correlation with end-of-study HRQoL	Positive mood		Negative mood		BI anxiety	
			Median (IQR)	Wilcoxon rank sum or Kruskal Wallis test *p*-value	Median (IQR)	Wilcoxon rank sum or Kruskal Wallis test *p*-value	Median (IQR)	Wilcoxon rank sum or Kruskal Wallis test *p*-value
Frequency								
Once	79.06	–0.023	4.0 (3.0–6.0)	< 0.001	4.0 (2.0–6.0)	0.185	1.0 (1.0–5.0)	< 0.001
Twice	17.19		7.0 (4.0–8.0)		4.0 (3.0–7.0)		5.0 (1.0–5.0)	
Three or more times	2.34		6.0 (4.0–8.0)		5.0 (4.0–6.0)		5.0 (2.0–5.0)	
Clean Intervals								
More than 4 h	54.8	–0.071	4.0 (4.0–6.0)	0.002	5.0 (4.0–7.0)	< 0.001	1.0 (1.0–2.0)	< 0.001
Less than 4 h	35.34		3.0 (3.0–6.0)		3.0 (2.0–4.0)		2.0 (1.0–5.0)	
Always soiled	9.77		2.0 (1.0–3.0)		5.0 (3.0–7.0)		5.0 (2.0–5.0)	
Amount								
Small	52.99	–0.470***	4.0 (3.0–6.0)	0.554	5.0 (2.0–6.0)	0.046	1.0 (1.0–5.0)	< 0.001
Medium	33.58		4.0 (3.0–8.0)		3.0 (2.0–5.0)		5.0 (1.0–5.0)	
Large	13.43		4.0 (3.0–6.0)		4.0 (3.0–7.0)		5.0 (5.0–5.0)	
Independent management								
No	19.40	0.571***	3.0 (2.0–4.0)	< 0.001	8.0 (4.0–10.0)	< 0.001	1.0 (1.0–5.0)	0.022
Yes	80.60		4.0 (4.0–7.0)		4.0 (2.0–5.0)		5.0 (2.0–5.0)	
Avoided any activities								
No	79.85	–0.286**	6.0 (4.0–8.0	0.002	4.0 (3.0–6.0)	0.210	1.0 (1.0–5.0)	< 0.001
Yes	20.15		4.0 (3.0–6.0)		4.0 (2.0–6.0)		5.0 (2.0–5.0)	

* *p*0.05;

** *p* < 0.01;

*** *p* < 0.001

*Supplementary analyses*. We conducted supplementary analyses to examine whether the daily frequency or context differed for UI as compared with BI. UI occurred significantly *more* often (median: twice vs once; *p* < 0.001), with significantly *greater* volume (*p* < 0.001) and significantly *shorter* dry intervals (*p* < 0.001) as compared with BI. YASB reported activity avoidance significantly *less often* on days with reports of UI as compared to with BI (*p* < 0.001). Positive mood was significantly *higher* and negative mood significantly *lower* on days with UI vs BI (both *p* < 0.001). YASB felt significantly *less anxious* about experiencing UI than they did BI (*p* < 0.001).

### Objective 2: Associations between daily UI and BI frequency and characteristics with daily mood and incontinence anxiety

*Urinary incontinence.* As indicated in Table II, median daily positive mood *decreased* significantly when YASB experienced more frequent UI, shorter intervals between UI events, larger amounts of UI, and when they avoided any activities because of UI (all *p* < 0.001). Median daily negative mood significantly *increased* when YASB reported more frequent UI, shorter time in between UI events, and larger amounts of UI (all *p* < 0.001). YASB median anxiety about UI was significantly *higher* in association with more frequent UI and shorter intervals between UI (both *p* < 0.001), as well as avoiding any activities due to UI (*p* = 0.002).

*Bowel incontinence.* As presented in Table III, median daily positive mood was significantly *lower* when YASB reported more frequent BI (*p* < 0.001), shorter intervals between BI events (*p* = 0.002), being able to manage BI independently (*p* < 0.001), and avoiding any activities because of BI (*p* = 0.002). Median daily negative mood significantly *increased* when YASB experienced shorter time in between BI events (*p* < 0.001), larger amounts of BI (*p* = 0.046), and having to have help to manage the BI event (*p* < 0.001). YASB median anxiety about BI was significantly higher with more frequent BI (*p* < 0.001), shorter intervals between BI events (*p* < 0.001), larger amounts of BI, needing help managing BI (*p* = 0.002), and avoiding any daily activities due to BI (*p* < 0.001).

### Exploratory objective: associations between daily UI and BI frequency and characteristics and end-of-study health related quality of life, and between daily mood, daily incontinence anxiety and end-of-study health related quality of life

*Urinary incontinence*. As revealed in Table II, more frequent daily UI (ρ = –0.253; *p* < 0.001), shorter time dry intervals (ρ = –0.277; *p* < 0.001), larger amount of UI (ρ = –.286; *p* < 0.001), and avoiding activities because of UI (ρ = –0.168; *p* < 0.001) were significantly correlated with lower end-of-study HRQoL.

*Bowel incontinence.* As indicated in Table III, larger amounts of BI (ρ = –0.470; *p* < 0.001) and avoiding activities because of BI (ρ = –0.286; *p* < 0.001) were significantly correlated with lower end-of-study HRQoL. Being able to independently manage BI events was associated with higher end-of-study HRQoL (ρ = 0.571; *p* < 0.001).

*Daily mood and daily incontinence anxiety*. Higher daily median mood was positively correlated with HRQoL (ρ = 0.222; *p* = 0.019), while higher daily median negative mood (ρ = –0.687; *p* < 0.001), higher daily UI anxiety (ρ = –0.506; *p* < 0.001) ≤ and higher daily median BI anxiety (ρ = –0.191; *p*-0.044) were significantly associated with lower HRQoL.

## DISCUSSION

Rehabilitation-focused continence care for YASB prioritizes reducing the intrusion of UI and BI into daily life. This requires characterizing when, where, and under what conditions incontinence occurs, because these contextual factors help explain differences both in same-day emotional distress and longer-term HRQoL (4, 17–20). This study is one of the first to systematically collect daily continence and emotion data among YASB using ecological momentary assessment. Our data demonstrate that incontinence does not affect all days – or all YASB – in the same way. We outline 2 implications of this variability for individualized rehabilitation management.

We observed 2 key forms of variability. First, both objective symptom characteristics (e.g., frequency and volume of incontinence) and subjective responses (e.g., activity avoidance, reduced positive mood, increased negative mood, and incontinence-specific anxiety) fluctuated across days. Second, these associations differed by incontinence type, such that the emotional correlates of urinary incontinence were not identical to those of bowel incontinence. Together, these findings demonstrate that incontinence burden is not stable across time; similar symptom profiles may have different emotional consequences depending on contextual and management factors, even within the same individual. Reliance on retrospective summaries of “typical” symptoms may therefore obscure meaningful day-level variation in mood, anxiety, and perceived quality of life. Rehabilitation strategies that do not account for this variability may be insufficiently responsive to patient lived experiences with incontinence. In contrast, approaches informed by repeated, in-the-moment assessment may better support individualized management. Integration of EMA data into intervention design, including just-in-time adaptive approaches described in other adolescent and young adult populations (37), may offer one strategy for applying these findings in rehabilitation care. Although such strategies have not yet been evaluated in SB, the present data suggest they warrant further investigation, particularly during periods of symptom fluctuation or between clinic visits.

The HRQoL findings extend the clinical relevance of these results. HRQoL was associated with day-level symptom characteristics and daily emotional responses, suggesting that repeated daily experiences may shape end-of-month perceptions of quality of life. Accordingly, rehabilitation assessment may benefit from incorporating attention to day-to-day symptom variability rather than relying solely on global summary ratings. These associations differed by incontinence type, with objective symptom characteristics more consistently related to HRQoL in UI and management demands more strongly linked to HRQoL in BI. Across both incontinence types, higher daily negative mood and greater incontinence-specific anxiety were associated with lower HRQoL, underscoring the importance of negative emotional burden for longer-term quality-of-life outcomes. Although HRQoL is often assessed infrequently and conceptualized as relatively stable, these findings suggest that short-term variability in characteristics and emotional responses is clinically relevant to longer-term quality-of-life outcomes. Exclusive reliance on retrospective HRQoL ratings may therefore overlook meaningful day-level fluctuations that occur between assessments.

In summary, clinical management of UI and BI focuses primarily on decreasing symptom severity by making incontinence less frequent and smaller in amount. As this paper shows, these are worthwhile clinical targets. Incontinence severity matters to YASB. Overall, YASB with more frequent UI and larger amounts of BI reported worse daily-reported mood and higher anxiety, and, corroborating previous reports in children and adults with SB, also reported lower HRQoL (8, 38). A novel finding in this study is that YASB who avoided activities because of either UI or BI also reported worse mood, higher anxiety, and lower HRQoL. This suggests that some incontinence interventions may still be effective even if they focus on mitigating the negative social consequences of incontinence once it occurs, rather than preventing incontinence itself. Additionally, independently managed BI, but not UI, was associated with better mood, less anxiety, and higher HRQoL. Therefore, improving self-management in BI care may be particularly beneficial for short- and long-term well-being in this population. Future work should further examine how daily incontinence experiences translate into longer-term HRQoL and further elucidate intervention targets that address both symptom burden and associated emotional distress.

### Limitations

This study has several methodological limitations. Although some measures (e.g., affect) have been used in prior EMA research, other measures were adapted from instruments previously validated in retrospective formats in this population (4, 17). While initial findings support the feasibility of these adaptations, additional psychometric evaluation is warranted in future studies. The 30-day assessment period may also have been insufficient to capture longer-term patterns or cyclical variability in UI and BI. Extended monitoring periods may provide a more comprehensive representation of incontinence patterns over time. Longer observation windows would also permit more detailed examination of contextual factors, such as self-management behaviours, pain, and hygiene demands, that may influence daily mood and incontinence-related anxiety. These considerations will inform the design of future investigations.

The current sample also has limitations. Participants were predominantly White, female, heterosexual, well educated, stably employed, and demonstrated relatively high levels of mobility and living independence. These characteristics may reflect self-selection bias, potentially limiting representation of individuals with greater caregiver dependence or more significant developmental delay. Although gender and neurological characteristics were generally consistent with other SB research samples, the findings may not generalize to more diverse populations. The parent study excluded individuals under 18 years of age; thus, the results do not capture earlier developmental stages during which continence management skills and emotional responses are established. Longitudinal research spanning adolescence through adulthood would help clarify how daily incontinence experiences relate to longer-term HRQoL trajectories. In addition, participants had a recent history of incontinence, and daily patterns may differ among YASB with less frequent symptoms. Replication in more diverse and clinically heterogeneous samples is warranted.

### Conclusion

This study joins an emerging field of prospective, day-level examinations of urinary and bowel incontinence among young adults with spina bifida. The findings demonstrate substantial day-to-day variability in both UI and BI symptom characteristics and emotional responses, with implications for same-day functioning and longer-term health-related quality of life. Given the increasing demands for independence and self-management during this life stage, attention to day-level variability may be particularly relevant for rehabilitation care. Collectively, the findings support consideration of continence management approaches that move beyond retrospective symptom summaries toward more dynamic and individualized monitoring strategies. EMA-based self-monitoring may facilitate collaborative patient–clinician evaluation (39) and support treatment plans (40) that can be iteratively and dynamically adjusted as needed (41).
